# 378. Visby Medical Women’s Sexual Health Test: An Over-the-Counter Molecular Diagnostics Solution to STI Testing *The Visby Medical Women’s Sexual Health test has not been cleared or approved by the FDA for in vitro diagnostic use

**DOI:** 10.1093/ofid/ofae631.009

**Published:** 2025-01-29

**Authors:** Shradha Prabhulkar, Jennifer Albrecht, Wendy Trinh, Brittany Owings, Sayani Nandy, Allie Buccino, Huda Mutwakil, Paul Dentinger, Gary Schoolnik, Beth Lingenfelter

**Affiliations:** Visby Medical, San Jose, CA; Visby Medical, San Jose, CA; Visby Medical, San Jose, CA; Visby Medical, San Jose, CA; Visby Medical, San Jose, CA; Visby Medical, San Jose, CA; Visby Medical, San Jose, CA; Visby Medical, San Jose, CA; Visby Medical, San Jose, CA; Visby Medical, San Jose, CA

## Abstract

**Background:**

Visby Medical has developed a palm-sized, single-use, PCR platform that combines sample preparation, nucleic acid amplification and detection into an integrated device (Figure 1) that delivers results within 30 minutes. This platform was used to develop the Women’s Sexual Health Test for the detection of DNA from pathogens *Chlamydia trachomatis*, *Neisseria gonorrhoeae* and *Trichomonas vaginalis*. Testing instructions were designed for lay users. A smartphone application was developed to guide users through the testing process with videos, including digital interpretation of the results using the smartphone camera.

**Table 1:**
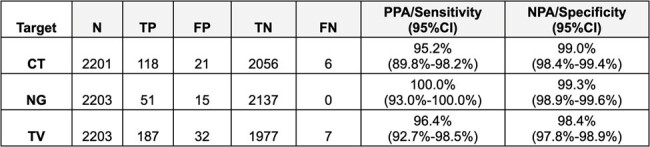
The data summary table below shows the aggregate results from testing conducted by lay users across all thirteen sites to assess the performance of the Visby test relative to the comparator tests. PPA=Positive Percent Agreement; NPA=Negative Percent Agreement. TP=true positive; FP=false positive; TN=true negative; FN=false negative.

**Methods:**

Performance characteristics of an investigational use only (IUO) version of the test were evaluated using clinical and analytical studies. The clinical study was carried out at 13 geographically diverse sites. A total of 2203 female subjects (14+ years old) were included in the performance evaluation. Subjects followed the printed and/or the digital instructions to self-collect a vaginal swab, place it in the Visby collection media, conduct the test, and interpret the results. Three additional swabs were collected from each subject for comparator testing. The analytical studies included limit of detection (LoD), inclusivity, cross reactivity, interference and flex studies. Human factors usability studies were also conducted.

**Figure 1:**
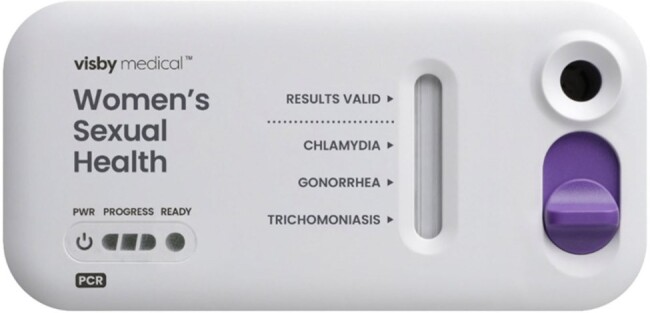
Visby Medical Women’s Sexual Health Test

**Results:**

The Visby Medical Women’s Sexual Health Test* had a sensitivity of at least 95.0% and a specificity of at least 98.0% (see Table 1) for each of the three target organisms. Human Factors usability studies substantiated that a lay user could successfully perform the testing, and the analytical studies demonstrated that the test is sensitive (LoD, inclusivity), specific (cross-reactivity), and robust (interference and flex testing).

**Conclusion:**

The performance characteristics of the Visby Medical Women’s Sexual Health Test when used by lay users in an at-home setting was equivalent to the results from high complexity assays, conducted in centralized laboratories by trained laboratory professionals. This test could potentially be the first PCR test cleared for at-home use with the ability to offer highly accurate testing for sexually transmitted infections (STIs) and remove barriers to promptly and accurately treating STIs.

**Disclosures:**

**Jennifer Albrecht, PhD**, Visby Medical, Inc: Employee **Brittany Owings, n/a**, Visby Medical: Employee **Paul Dentinger, PhD**, Visby Medical: Stocks/Bonds (Private Company) **Gary Schoolnik, MD**, Visby Medical: I am the Chief Medical Officer of Visby Medical|Visby Medical: Stocks/Bonds (Private Company) **Beth Lingenfelter, n/a**, Visby Medical: Employee

